# Analytical Characterization, Pharmacokinetics, Safety, and Preliminary Immunogenicity of GNR-062, an Agalsidase Beta Biosimilar Candidate, in Healthy Male Volunteers

**DOI:** 10.3390/biotech15040094

**Published:** 2026-10-14

**Authors:** Ivan Lyagoskin, Maksim Smolov, Oksana Markova, Vladislav Klyashtorny, Marina Pantyushenko, Mariya Koroleva, Olga Strizhakova, Rakhim Shukurov, Ravil Khamitov

**Affiliations:** Joint Stock Company GENERIUM, 14, Vladimirskaya Street, Volginskiy 601125, Vladimir Region, Russiaoamarkova@generium.ru (O.M.); vgklyashtornyj@generium.ru (V.K.); pantushenko@ibcgenerium.ru (M.P.); makoroleva@ibcgenerium.ru (M.K.); strizhakova@ibcgenerium.ru (O.S.); shukurov@ibcgenerium.ru (R.S.); khamitov@ibcgenerium.ru (R.K.)

**Keywords:** Fabry disease α-galactosidase A, agalsidase β, Fabrazyme^®^, biosimilar enzyme replacement therapy, biosimilar, pharmacokinetics, immunogenicity, clinical trial

## Abstract

Fabry disease is a rare genetic multisystemic lysosomal disease characterized by progressive cellular accumulation of globotriaosylceramide (GL-3) in various organs with specific cutaneous (angiokeratoma), neurological (pain), renal (proteinuria, chronic kidney failure), cardiovascular (cardiomyopathy, arrhythmia), cochleovestibular and cerebrovascular manifestations (transient ischemic attacks, strokes). The phenotypic expression depends on the age of onset and, in females, on the level of X-inactivation. Enzyme replacement therapy is currently the standard therapy for the disease. JSC GENERIUM recently developed a potential biosimilar of Fabrazyme^®^, GNR-062, and investigated its physicochemical and biological properties, pharmacokinetics and immunogenicity to assess its bioequivalence to Fabrazyme^®^. Direct evaluation of the N-glycosylation profile by HPLC revealed differences mainly related to the distribution of complex sialylated glycans. The overall sialylation level was comparable between the two products. The same conclusion follows from the results of direct analysis of the sialic acid content. Despite a small difference of a few percent in biphosphorylated glycan content, the overall measured M6P values were comparable. Thus, the internalization of agalsidase β into the inner space of lysosomes by binding to key receptors appear to be the same for both products. In a single-blind, randomized comparative study of the pharmacokinetics and safety of a single intravenous infusion of GNR-062 (JSC GENERIUM, Russia) and Fabrazyme^®^ (Genzyme Europe B.V., The Netherlands) in a crossover design in healthy volunteers, the ratio of geometric means (90% CI) of AUCinf and Cmax for GNR-062 to agalsidase β was 0.9736 (0.9413–1.0070) and 0.9867 (0.9546–1.0199), respectively. These studies demonstrated pharmacokinetic bioequivalence of GNR-062 to agalsidase β. Immunogenicity findings are preliminary, as the study was not powered for formal immunogenicity equivalence, and the single-dose design in healthy volunteers does not reflect repeated long-term administration in patients with Fabry disease. In conclusion, GNR-062 demonstrated acceptable short-term tolerability in healthy volunteers and warrants further investigation as a potential therapeutic option for patients with Fabry disease.

## 1. Introduction

Fabry disease, or Anderson–Fabry disease, is a hereditary disorder belonging to the group of lysosomal storage diseases caused by decreased or absent activity of the enzyme α-galactosidase A. This enzyme deficiency leads to the accumulation of glycophospholipids in lysosomes of cells in various organs, including the heart, kidneys, nervous system, and vascular endothelium. The disease is progressive and multisystemic, characterized by a decrease in quality of life and life expectancy. Without appropriate treatment, death typically occurs in the fourth to fifth decade of life due to cardiovascular or cerebrovascular complications or renal failure [1,2]. Disease severity correlates with residual α-galactosidase A activity and is diagnosed by decreased activity in leucocytes, plasma, or cultured dermal fibroblasts [3]. The estimated incidence of Fabry disease is approximately 1 in 50,000 males, with a prevalence ranging from 0.85 to 2.5 cases per 100,000 individuals worldwide, making it the second most common lysosomal storage disease after Gaucher disease. Mortality increases with age, often resulting from renal, cardiac, or cerebrovascular complications during the fourth or fifth decade of life. Females who are heterozygous for the classic phenotype can range from being asymptomatic to as severely affected as homozygous males [4,5]. Current treatment includes enzyme replacement therapy (ERT) and pharmacological chaperone therapy. Two α-galactosidase A formulations are approved for ERT: agalsidase α (Replagal^®^, Shire) and agalsidase β (Fabrazyme^®^, Genzyme). These therapies reduce GL-3 accumulation in target tissues, alleviate pain, and help stabilize renal function [6].

GNR-062 is a potential biosimilar of agalsidase β developed in Chinese hamster ovary cells that shares an identical primary amino acid sequence with the reference product. Analytical studies have demonstrated a similar charge isoform distribution as well as enzymatic activity and cellular uptake capacity, while the glycosylation profile appears to be slightly different for N-glycans carrying sialic acids and/or mannose-6-phosphate moieties. The described Phase I clinical study in Russia aimed to evaluate the bioequivalence of GNR-062 to agalsidase β by assessing its pharmacokinetics, and safety in healthy adults.

## 2. Materials and Methods

A Phase I clinical trial (AGS-FBD-I, v3, 05.07.2023) was devoted to the study of the bioequivalence of GNR-062 and Fabrazyme^®^, including the evaluation of safety and pharmacokinetics. The trial was conducted in compliance with the principles of the Declaration of Helsinki.

All procedures and the informed consent form were approved by an institutional review board, and informed consent was obtained from each participant.

### 2.1. Materials

GNR-062 (JSC GENERIUM, Volginskiy, Russia) is identical to Fabrazyme^®^ (Genzyme Europe B.V., Waterford, Ireland) in both active ingredient (5 mg/mL agalsidase β) and excipients (mannitol, sodium dihydrogen phosphate monohydrate, sodium hydrogen phosphate heptahydrate). Four biosimilar batches manufactured over one year were compared with four EU-sourced commercial batches of the reference product with varying expiration dates.

Trimethylamine, butylamine, sodium phosphate, sodium chloride, bovine serum albumin (BSA), magnesium chloride, calcium chloride, N-acetylneuraminic acid, N-glycolylneuraminic acid, boric acid and 4-nitrophenyl-α-D-galactopyranoside were purchased from Sigma-Aldrich (Burlington, MA, USA). Gibco Dulbecco’s Modified Eagle Medium: Nutrient Mixture F12 (DMEM F12) medium was purchased from Fisher Scientific (Waltham, MA, USA). Acetic acid, citric acid, di-Sodium Hydrogen Phosphate 7-hydrate and phosphoric acid were purchased from PanReac AppliChem (Darmstadt, Germany). HPLC solvents as well as acetonitrile, tetrahydrofuran, and methanol were purchased from J.T. Baker (Phillipsburg, NJ, USA). D-Mannose 6-phosphate sodium salt was purchased from (Santa Cruz Biotechnology, Dallas, TX, USA). 4-nitrophenol was purchased from (Merck, Rahway, NJ, USA). Triton X-100 solution (Thermo Fisher Scientific, Waltham, MA, USA) and Protease Inhibitor Cocktail (Sigma-Aldrich, Burlington, MA, USA) were also purchased. Phosphate-buffered saline (PBS) was purchased from PanEco (Moscow, Russia). 95% ethanol was purchased from AO RFK (Moscow, Russia). Human fibroblasts (GM00107, Coriell Institute, Camden, NJ, USA) were used.

Pharmacokinetic and anti-drug antibody assays were performed on 96-well plates (MaxiSorp, Nunc, Roskilde, Denmark). Polyclonal rat antibodies against Fabrazyme^®^ were produced at GENERIUM JSC. Naglazyme^®^ (BioMarin International Limited, Dublin, Ireland) was used to assess the specificity of the method for determining agalsidase β concentration. The HRP conjugate of polyclonal rat antibodies was prepared with the LYNX Rapid Horseradish Peroxidase Antibody Conjugation Kit (Bio-Rad, Hemel Hempstead, Hertfordshire, UK), and biotinylated agalsidase β (GNR-062) was prepared using the Biotin Fast Conjugation Kit (Abcam, Cambridge, UK). The anti-drug antibody assays also used horseradish peroxidase (HRP)-conjugated streptavidin (Abcam, Cambridge, UK); Natural human IgG protein (Abcam, Cambridge, UK), Natural human IgM protein (Abcam, Cambridge, UK), Natural human IgE protein (Abcam, Cambridge, UK), Mouse monoclonal [JDC-10] Anti-Human IgG Fc (HRP) (Abcam, Cambridge, UK), Anti-IgM antibody [CH2] (HRP) (Abcam, UK), Anti-IgE antibody [1a2] (HRP) (Abcam, Cambridge, UK). Tetramethylbenzidine (TMB) was purchased from Vektor Corporation (Marion, IA, USA).

### 2.2. N-Glycans Profiling

Glycosylation microheterogeneity was characterized by high-performance liquid chromatography (HPLC) with fluorescence detection. Glycans were enzymatically released from the test sample using PNGase F and subsequently labeled with the Instant AB fluorescent dye, employing the GlycoPrep^®^ Rapid N-Glycan Preparation platform (Agilent Technologies, Santa Clara, CA, USA). The labeled glycan mixtures were separated using an inert Acquity Arc Bio System (Waters, Milford, MA, USA) equipped with an Asahipak NH2P-50 4E column (4.6 × 250 mm, 5 µm, 100 Å (Shodex, New York, NY, USA). Separation was achieved with a mobile phase consisting of an aqueous solution of 6% acetic acid and 6% triethylamine (*v*/*v*), using a 60-min decreasing linear gradient of acetonitrile from 75% to 0%. The flow rate was maintained at 0.8 mL/min, and the column temperature was set to 50 °C. Fluorescence detection was performed with excitation and emission wavelengths of 285 nm and 345 nm, respectively. Data acquisition and processing were conducted using Empower™ 3 software (Waters, Milford, MA, USA).

### 2.3. Monosaccharide Content

Monosaccharide composition was determined using a commercial kit (Monosaccharide Release and Labeling Kit, Ludger Ltd., Oxfordshire, UK). Briefly, each sample was hydrolyzed with 6.7 M trifluoroacetic acid at 100 °C for 1.5 h. The hydrolysates were then dried under vacuum, reconstituted in water, and derivatized with a solution of 0.1 M anthranilic acid and 0.3 M sodium cyanoborohydride in methanol containing 2.4% sodium acetate and 2% boric acid. Derivatization was performed at 80 °C for 45 min. The resulting samples were analyzed using an Alliance e2695 HPLC system (Waters, Milford, MA, USA) equipped with a 2475 fluorescence detector and a Symmetry^®^ C18 column (3.9 × 150 mm, 5 µm, 100 Å; Waters, Milford, MA, USA). The mobile phase consisted of an aqueous solution of 0.2% butylamine, 0.5% phosphoric acid, and 1% tetrahydrofuran (*v*/*v*), adjusted to pH 1.9, with an acetonitrile gradient of 2.5% to 10% over 40 min. The separation was carried out at a flow rate of 0.5 mL/min and a column temperature of 30 °C. Fluorescence detection was performed with excitation and emission wavelengths of 360 nm and 425 nm, respectively. Quantification was achieved by external calibration using standard monosaccharides, and data were processed with Empower™ 3 software (Waters, Milford, MA, USA). The results were normalized to the enzyme content and were expressed as mol of monosaccharide per mol of protein dimer.

### 2.4. Sialic Acids

The sialic acid content was determined using the LudgerTag™ DMB Sialic Acid Release and Labeling Kit (Ludger Ltd., Oxfordshire, UK). Briefly, samples were dissolved in 25 mM Tris-HCl buffer pH 7.5 and hydrolyzed with 2 M acetic acid at 80 °C for 2 h. After cooling to room temperature, the hydrolysates were derivatized with 1,2-diamino-4,5-methylenedioxybenzene (DMB) at 50 °C for 3 h in the dark. The derivatized samples were analyzed using an ACQUITY UPLC H-Class system (Waters, Milford, MA, USA) equipped with a Symmetry^®^ C18 column (3.9 × 150 mm, 5 µm, 100 Å; (Waters, Milford, MA, USA). Isocratic elution was performed with a mobile phase consisting of water, acetonitrile (9% *v*/*v*), and methanol (7% *v*/*v*) at a flow rate of 0.5 mL/min and a column temperature of 30 °C. Fluorescence detection was carried out with excitation and emission wavelengths of 373 nm and 448 nm, respectively. Quantification was performed by external calibration using defined standards of N-acetylneuraminic acid (Neu5Ac) and N-glycolylneuraminic acid (Neu5Gc). Data were processed using Empower™ 3 software (Waters, Milford, MA, USA). The results were normalized to the active dimer content and were expressed as mol of sialic acid per mol of protein.

### 2.5. Cellular Uptake

M6PR-dependent cellular uptake of the enzymes was examined using Fabry disease patient fibroblasts, which were seeded onto 96-well plates at a density of 3.0 × 10^4^ cells/well and cultured for 4 days. Varying concentrations of GNR-062 or Fabrazyme^®^ were added to the culture medium, followed by incubation at 37 °C for 5 h. The cells were washed with phosphate-buffered saline and then lysed with 1% Triton X-100 solution containing 0.5% Protease Inhibitor Cocktail, then the cells were frozen/thawed three times, while monitoring the level of lysis under a microscope. Concentrations of GNR-062 or Fabrazyme^®^ in the cell lysates were measured based on an enzyme activity assay using 4-nitrophenyl-α-D-galactopyranoside (pNP-GAL) as a substrate. To assess the specificity of internalization through mannose-6-phosphate receptors, separately isolated wells were used with equal concentrations of agalsidase β (1000 ng/mL), with and without the addition of mannose-6-phosphate (5 mg/mL) (D-Mannose 6-phosphate sodium salt). The number of biological replicates was 4, and the number of technical replicates was 3. Independent experiments were performed on 3 occasions. A four-parameter logistic model was fitted to the optical density data using GraphPad Prism 8 (GraphPad Software, San Diego, CA, USA). The specific activity of each sample was measured as a percentage relative to the original Fabrazyme^®^ product, which was selected as the standard. EC_50_ values were estimated with 95% CI. Relative potency was calculated as the ratio of EC_50_ values (Fabrazyme/GNR-062) with 78% CI.

### 2.6. Enzyme Activity

The method was based on the enzymatic hydrolysis of the synthetic substrate 4-nitrophenyl-α-D-galactopyranoside (pNP-α-D-GAL), which produces a colored product: 4-nitrophenol. The amount of enzyme required to hydrolyze 1 µmol of substrate per minute under the assay conditions is defined as one unit of activity. The test samples were diluted to a protein concentration of 0.15–0.5 μg/mL in a reaction buffer (citric acid-sodium phosphate buffer, pH 4.6). 4-nitrophenol was diluted to a concentration of 0.35 μmol/mL in the reaction buffer. The substrate 4-nitrophenyl-α-D-galactopyranoside was dissolved in the reaction buffer containing 2% ethanol. Sample dilutions, 0.35 μmol/mL 4-nitrophenol solution (standard solution) and the reaction buffer (reference solution) were pipetted into wells of a 96-well plate. The reaction was started by the addition of the substrate solution. The plate was incubated in a thermostatic shaker for 20 min at 37 °C. The reaction was stopped by the addition of borate buffer, pH 9.0, to the wells. The amount of 4-nitrophenol in the wells was determined spectrophotometrically at 405 nm. The number of biological replicates was 4, and the number of technical replicates was 4. Independent experiments were performed on 3 occasions.

### 2.7. Phase I Clinical Trial

#### 2.7.1. Participant Characteristics

The study enrolled healthy male volunteers aged 18–41 years, weighing 50–70 kg (body mass index [BMI] 18.5–29.9 kg/m^2^). Each participant provided written informed consent. Key exclusion criteria comprised: a history of vital organ dysfunction (including, but not limited to, hepatic, renal, and cardiovascular impairment); the presence of antibodies to hepatitis C virus, a positive rapid plasma reagin/Treponema pallidum chemiluminescence assay, and/or HIV-1/2 infection; current or past alcohol abuse; previous treatment with α-galactosidase A agents (agalsidase α or β); and other criteria at the discretion of the investigator [7].

#### 2.7.2. Trial Design

A single-blind, randomized comparative study of the pharmacokinetics and safety of a single intravenous infusion of GNR-062 (JSC GENERIUM, Russia) and Fabrazyme^®^ (Genzyme Europe B.V., Amsterdam, The Netherlands) in a crossover design in healthy volunteers at a dose of 1 mg/kg (AGS-FBD-I, v3, 05.07.2023). The study was approved by the Independent Ethics Committee at the I.M. Sechenov First Moscow State Medical University of the Ministry of Health of the Russian Federation (Sechenov University), approval date: 26 January 2023. The test or reference drug was administered at a dose of 1 mg/kg body weight as an intravenous infusion lasting 300 min.

The washout period between the two infusions was 14 days (Day 1 to Day 15). This duration was selected based on the known pharmacokinetic profile of agalsidase β (T1/2 ≈ 45–90 min) and the expectation that the drug would be completely cleared from the circulation within 24 h.

This study was single-blind, meaning the volunteer did not know which infusion they were receiving. The investigator was informed of the drug the volunteer was receiving after randomization on Day 1.

Processing and analysis of all PK and ADA samples were performed by laboratory personnel blinded to the treatment allocation.

The objectives of the study were: 1. comparative assessment of the safety and tolerability of the drugs GNR-062 and Fabrazyme^®^ in terms of incidence, severity, and severity of adverse events (AEs), and the cause-and-effect relationship of AEs with the use of the study drugs during the study period according to subjective complaints, physical examination, assessment of vital signs, and laboratory and instrumental studies of volunteers in the comparison groups; 2. to prove the equivalence of the drug GNR-062 at a dose of 1 mg/kg to the drug Fabrazyme^®^ at a dose of 1 mg/kg with a single intravenous infusion to healthy volunteers according to the pharmacokinetic parameters AUC and AUCinf,; 3. to assess and compare the pharmacokinetic parameters (AUC, AUCinf, Cmax, Tmax, T1/2, Kel, CL, Vd) of the drug GNR-062 at a dose of 1 mg/kg and the drug Fabrazyme^®^ at a dose of 1 mg/kg as a single intravenous infusion to healthy volunteers; 4. to compare the frequency of formation of anti-drug antibodies after a single intravenous infusion of GNR-062 at a dose of 1 mg/kg and Fabrazyme^®^ at a dose of 1 mg/kg, their titer and neutralizing activity.

#### 2.7.3. Study Endpoints

The primary endpoint was bioequivalence between GNR-062 and Fabrazyme^®^, evaluated by measuring serum α-galactosidase A levels in a laboratory and calculating pharmacokinetic parameters (see Section 2.8). AEs, ADRs, clinical and physical examinations, 12-lead electrocardiograms, and vital signs were assessed at predefined time points. Serious AEs were defined as death or events that might lead to death, persistent dysfunction or disability, or necessitate hospitalization. Anaphylactic shock, IARs, and other non-serious AEs capable of causing study discontinuation or notable changes in clinical laboratory examinations were designated as important AEs and monitored closely. Antibodies against α-galactosidase A in sera were assessed in blood collected before and after the trial and at the follow-up visit.

The causality of each AE was assessed by the investigator using predefined criteria: temporal relationship to infusion, biological plausibility, known effects of the drug class, and alternative explanations. AEs were classified as unrelated, unlikely, possibly, probably, or definitely related.

#### 2.7.4. Statistical Analysis

##### Analytical Similarity

For physicochemical parameters, the acceptance range was defined as the reference product mean ± 3 standard deviations (M ± 3 SD), which corresponds to a 99.7% tolerance interval under the assumption of normality. For biological attributes (e.g., cellular internalization, enzymatic activity) that followed a log-normal distribution, values were log-transformed (base 10), and the acceptance range was calculated as log(M) ± 2 SD of the log-transformed values (i.e., M ×/÷ SD^2^ on the original scale). The use of ± 2 SD for biological attributes reflects their higher inherent variability and is consistent with the risk-based approach described in [8,9,10]. With only four batches per product, estimates of mean and SD are associated with uncertainty; therefore, batch-level results and assay variability were reported alongside the acceptance ranges.

##### Bioequivalence Analysis

Analysis populations. Bioequivalence was analyzed in the pharmacokinetic set (PKs), which included all randomized participants who received at least one dose of the study drug and had at least one post-dose serum concentration of agalsidase β measured. The per-protocol (PP) population, used for the primary confirmatory analysis, comprised participants who completed both treatment periods without major protocol deviations. The safety set (SS) included all participants who received at least one dose of the study drug. The full analysis set (FAS) included all randomized participants.

Pharmacokinetic parameters. Serum concentrations of agalsidase β were used to determine the following pharmacokinetic parameters for each participant by non-compartmental analysis using PKSolver: AUCinf—area under the serum concentration–time curve from time 0 extrapolated to infinity (ng·min/mL); AUC0–t—area under the serum concentration–time curve from time 0 to the time of the last measurable concentration (ng·min/mL); Cmax—maximum serum concentration (ng/mL); Tmax—time to reach Cmax (min); T1/2—terminal elimination half-life (min); Kel—terminal elimination rate constant (min^−1^); CL—total body clearance (mL/min), calculated as Dose/AUCinf for intravenous administration; and Vd—apparent volume of distribution (L). Because the study drug was administered intravenously, no bioavailability correction (F) was applied; consequently, CL and Vd were reported without the “/F” notation.

Statistical model. The primary PK parameters (AUCinf, AUC0–t, and Cmax) were natural log-transformed prior to analysis. A mixed-effects model was fitted to the log-transformed data with treatment (GNR-062 vs. Fabrazyme), period, and sequence as fixed effects, and subject nested within sequence as a random effect. The two one-sided tests (TOST) procedure was applied to the differences in least-squares means of the log-transformed parameters. The geometric mean ratio (GMR) and its 90% confidence interval (CI) were obtained by back-transformation. Bioequivalence was concluded if the 90% CI for the GMR fell entirely within the acceptance range of 0.80–1.25, as recommended in the Guideline on the investigation of bioequivalence [11]. Summary statistics, geometric means, and geometric coefficients of variation (CVs) were calculated for each treatment group.

Handling of the incomplete subject. One randomized subject withdrew informed consent on Day 7, after completing Period I but before receiving the Period II infusion. This subject was included in the safety set (*N* = 36) and contributed Period I data to the descriptive PK summary. The primary confirmatory bioequivalence analysis was performed on the PP population (*N* = 35), comprising subjects who completed both periods, as pre-specified in the statistical analysis plan. The reason for discontinuation was withdrawal of informed consent and was not related to the study drug.

Assessment of carryover. Predose serum concentrations of agalsidase β on Day 15 (before the second infusion) were below the lower limit of quantification (LLOQ = 5 ng/mL) in all subjects who completed both periods. This confirmed that the 14-day washout period was sufficient and that no carryover effect was present.

Software. Statistical analysis of the PK parameters and processing of the concentration–time curves were performed using Stata 14 (StataCorp LLC, College Station, TX, USA) and PKSolver.

##### Immunogenicity Analysis

The incidence of anti-drug antibody (ADA) formation was compared between treatment groups using the chi-square test, or Fisher’s exact test when expected cell frequencies were below 5. For each group, the number and proportion of volunteers who developed ADAs were reported. Immunogenicity assessment parameters comprised the frequency of ADA formation, ADA titer, and the neutralizing activity of ADAs.

##### Safety Analysis

Safety was analyzed in the safety set (SS), which included all participants who received at least one dose of the study drug. Summary statistics were calculated for adverse events (AEs) and background characteristics. AEs were coded using the Medical Dictionary for Regulatory Activities (MedDRA) and summarized by system organ class (SOC) and preferred term (PT).

#### 2.7.5. Sample Size Determination

The study was designed to compare PK parameters of GNR-062 (1 mg/kg) and Fabrazyme^®^ (1 mg/kg) following a single intravenous infusion in healthy volunteers. The primary parameters were Cmax and AUC. Based on available literature data, the maximum coefficient of variation for these PK parameters was 27.6%. The study was designed to demonstrate that the 90% CI for the ratio of geometric means of the corresponding PK parameters for the two drugs fell within the range of 0.80–1.25. Sample size calculation was performed using PASS v.15 (Number Cruncher Statistical Systems, NCSS). It was calculated that at least 33 volunteers would be required to achieve 80% power in a 2 × 2 crossover design. To allow for a possible 10% early attrition rate, 36 subjects (18 per sequence) were planned to be randomized. Taking into account a possible 25% dropout rate during screening and 4 backup volunteers, 52 volunteers were planned to be screened.

### 2.8. Bioanalytical Methods

#### 2.8.1. Serum Sample Preparation and Storage

Blood samples for PK and ADA analysis were collected into clot activator tubes. To obtain serum, the blood was incubated at room temperature for 30 min and then centrifuged at 3500 rpm. Subsequently, 300 µL aliquots of serum were transferred into Eppendorf tubes and stored at −70 °C.

Blood samples for PK analysis were collected on Days 1 and 15: 5–15 min before infusion; 15, 30, 60, 90, and 180 min (±5 min) after the start of infusion; at 300 min (±5 min) (end of the 5-h infusion); and then 15, 30, 45, and 60 min (±5 min) and 90, 120, 180, and 420 min (±10 min) after the end of infusion.

For immunogenicity assessment, blood samples were collected before infusion on Days 1 and 15, with an additional sample obtained at the Day 29 follow-up visit.

#### 2.8.2. Pharmacokinetic Analysis

Agalsidase β serum levels were quantified by a validated enzyme-linked immunosorbent assay (ELISA). Polyclonal rat antibodies against Fabrazyme^®^ were used as the capture antibodies. The same antibodies conjugated to horseradish peroxidase (HRP) were used to detect the formed immune complexes. Fabrazyme^®^ was used to prepare the calibration standards. 3,3′,5,5′-Tetramethylbenzidine was used as the chromogenic substrate for HRP. The reaction was stopped by adding a stop solution, 0.5 M sulfuric acid, and absorbance was measured using a microplate spectrophotometer at a wavelength of 450 nm. The agalsidase β concentration in the test samples was derived from a calibration curve generated using a 4-parameter fit. All calculations were performed automatically using Bio-Rad software 6.3. (Microplate Manager, Herculane, California, USA).

The ELISA method was validated according to guideline ICH M10 and FDA [12,13,14]. The analytical range of the method was 5 to 5000 ng/mL for agalsidase β. The lower limit of quantification was 5 ng/mL (calculated as the LLOQ of 0.25 ng/mL multiplied by the minimum required 1:20 dilution). Based on all calibration standards, the mean inter-assay accuracy (% relative error) was 91.6 to 106.0%, and the mean inter-assay precision (% CV) was 2.0 to 12.5%. Inclusive of six quality control (QC) samples, the mean intra-assay precision ranged from 0.8 to 10.7%, the mean inter-assay accuracy ranged from 81.6 to 97.9%, the mean inter-assay precision ranged from 2.5 to 5.8%, and the total error (mean inter-assay accuracy plus mean inter-assay precision) ranged from 4.6 to 24.2%. Selectivity (matrix effect) was assessed using quality control (QC) samples prepared from individual sera of 40 healthy donors; for 80% of the samples, recovery (%R) was within the 80–120% range. The specificity of the method was confirmed using QC samples containing N-acetylgalactosamine-4-sulfatase (Naglazyme^®^). The stability of the control samples was demonstrated during storage for 30 days at temperatures not exceeding −20 °C, followed by transfer to temperatures not exceeding −70 °C and storage for 6 months.

#### 2.8.3. Anti-Drug Antibody Analysis

Immunogenicity assessment followed a multi-tiered strategy: screening, confirmation, titer determination for confirmed positive samples, and antibody class identification. The minimum required dilution (MRD) of serum samples for all assays was 1:5. Due to the unavailability of human antibodies to agalsidase β, rat polyclonal antibodies were used as a positive control in the total ADA assessment, which was acceptable according to FDA guidelines [15]. Furthermore, the species origin of the control was not critical for the bridging ELISA, as detection in this format did not require anti-species antibodies. Pooled serum from healthy donors (*N* = 50) served as the negative control. A sample was considered seronegative if the screening ELISA signal was below the cut-point or above the cut-point but not confirmed in the confirmatory assay.

In the screening assay, serum samples were tested for antibodies to agalsidase β (GNR-062) by bridging enzyme-linked immunosorbent assay (ELISA). GNR-062 was used as the capture reagent. Captured antibodies were eluted by acid dissociation and transferred onto another plate for adsorption. Detection was carried out using biotinylated agalsidase β (GNR-062) and HRP-conjugated streptavidin. TMB was used for visualization. The reaction was stopped by adding a stop solution, and optical density (OD) was measured at 450 nm using an xMark spectrophotometer (Bio-Rad, Hercules, CA, USA).

The confirmatory assay followed the same format as the screening assay, except for an additional competitive inhibition step with unlabeled drug: an excess of unlabeled agalsidase β (GNR-062) was added to the serum dilution buffer. The titer determination method was also similar to the screening assay but included serial dilution of samples.

Antibody classes (G, M, and E) were determined using mouse antibodies against the corresponding human immunoglobulin class conjugated to horseradish peroxidase (Abcam, UK). Natural human IgG, IgM, and IgE proteins directly adsorbed onto the plate (Abcam, UK) were used as positive controls.

Screening and confirmatory assay cut-points were determined parametrically using non-specific binding data from agalsidase β—naive serum samples. The cut-point factor (CPf_ST) for calculating the screening assay cut-point and determining the titer was 1.158; the cut-point (CP CT) for the confirmatory assay was 21.3%. The cut-point factors for the IgG, IgM, and IgE ADA assays (CPf_IgG, CPf_IgM, and CPf_IgE) were 1.074, 1.814, and 0.004, respectively. For the screening assay and the IgG and IgM ADA assays, the cut-point was calculated as the negative control optical density multiplied by the cut-point factor. For the IgE ADA assay, the cut-point was calculated as the negative control optical density plus the cut-point factor. The lower limit of detection for total ADA in the screening assay was 83 ng/mL. In the total ADA screening assay, intra-assay precision for positive controls at 10, 2, and 0.5 µg/mL ranged from 3.7% to 9.4%, while inter-assay precision ranged from 7.7% to 20.9%. The stability of control samples was confirmed during storage for 14 days at temperatures not exceeding −20 °C, followed by transfer and storage for 2 months at temperatures not exceeding −70 °C.

## 3. Results

### 3.1. Glycosylation

The amino acid sequence of recombinant agalsidase β contains N-glycosylation sites at positions 108, 161 and 184 [16]. Evidence that these sites are extensively modified comes from a significant (approximately 10 kDa) shift in the protein’s electrophoretic mobility upon selective deglycosylation [17]. In addition, the consensus sequence Asn-Pro-Thr corresponds to position 377, but in CHO cells this site is usually weakly modified [18]. Figure 1 shows representative chromatograms from the analysis of two drug products.

The typical glycan profile, consistent with previously published data [16,19,20,21], demonstrates the peaks corresponding to individual glycans or groups of glycans. These can be broadly categorized into regions for neutral (N), sialylated (S1–S3), and phosphorylated (P1, P2) forms. The neutral N-glycan group is composed predominantly of high-mannose and, to a lesser extent, biantennary complex structures. Although the total neutral glycan content is comparable between the two drug products, high-mannose species are relatively less abundant in GNR-062. Sialylated glycans containing N-acetylneuraminic acid residues are separated into peak groups S1–S3 based on the degree of branching in the polyantennary complex structures. This sialylation is known to directly influence agalsidase β characteristics, such as surface charge and three-dimensional conformation, thereby affecting its biological activity [17]. Despite observed product-specific variations, both drug products are characterized by a high total sialylated glycoforms content of approximately 40% (Table 1).

Sialylated and high-mannose N-glycans can influence the pharmacokinetics and pharmacodynamics of biomolecules. However, physiologically relevant differences typically arise from substantial variations in their content not observed in this study [22,23].

Oligosaccharides with terminal M6P residues play a key role in the mechanism of agalsidase β action. These residues mediate the internalization of the enzyme into the lysosomal compartment via active transport, which occurs through specific interactions with cellular receptors and components: MBL, MMR [16,24].

The proposed drug product GNR-062 is characterized by the pronounced abundance of mono- and bisphosphorylated high-mannose glycans with a total content of approximately 45%. This value is within a few percentage points of the level measured for the Fabrazyme^®^ reference product (Table 1). Despite an observed 3% discrepancy for bisphosphorylated species (P2) orthogonal assessment of the total M6P content demonstrated a reasonable comparability between the two products (Table 2).

N-glycans produced by CHO cell lines have the potential to increase immunogenicity. For this reason, oligosaccharides containing non-human residues, such as N-glycolylneuraminic acid (Neu5Gc) and the galactose-α-1,3-galactose (α-Gal) motif, are of particular concern [25]. However, analysis of the agalsidase β samples revealed no detectable level of these immunogenic structures. A separate orthogonal assessment confirmed that Neu5Gc was present only at an extremely low level. Furthermore, other monosaccharide composition values calculated for the active dimeric form showed good agreement with published data for Fabrazyme^®^ (Table 2) and indicated only minor differences for GNR-062 [16,17,19,26,27].

**Table 2:** Monosaccharide composition (mol/mol dimer).

Monosaccharide	Published Data [16,17,19,26,27]	Fabrazyme^®^	GNR-062
GlcNAc	7.9–18.4	15.8 ± 1.0	17.1 ± 1.1
GalNAc	‒	<0.5	<0.5
Galactose	6.4–8.3	6.3 ± 0.3	7.4 ± 0.3
Mannose	16.3–25.7	20.3 ± 1.1	19.4 ± 0.8
Glucose	‒	1.9 ± 0.2	2.2 ± 0.6
M6P	2.9–3.6	3.4 ± 0.3	3.2 ± 0.2
Fucose	1.8–2.1	2.0 ± 0.1	2.3 ± 0.2
NANA	5.5–7.3	5.6 ± 0.3	4.9 ± 0.5
NGNA	‒	<0.1	<0.1

### 3.2. Enzyme Activity of Agalsidase β

The specific agalsidase β activity values were comparable to that of Fabrazyme, with a mean of 69.6 U/mg (78% CI: [64.7–76.7]), which was within the predefined margin of M ± 2SD. The number of independent experiments was 3, with 4 technical replicates per experiment. Batch-level data are shown in Figure 2.

### 3.3. Penetration of Agalsidase β into Cells

M6PR-dependent cellular uptake of the enzymes was examined using Fabry disease patient fibroblasts (GM00107, Coriell Institute, Camden, NJ, USA). The uptake of GNR-062 was comparable to that of Fabrazyme, with a relative potency (EC_50_ ratio) of 108.8% (78% CI: [93.8–135.3]), which was within the predefined margin of M ± 2SD. The number of independent experiments was 3, with 3 technical replicates per experiment. Batch-level data are shown in Figure 3b. The dependence of the process on M6PR was confirmed by the reduction in this effect upon the addition of excess M6P (Figure 3c).

### 3.4. Results of Clinical Trials

#### 3.4.1. Study Design Outline

The study included: 1. Screening Period (Day -14 to Day -1); 2. Randomization—Day 1 (Visit 1); 3. Period I (Day 1–Day 14) including: Day 1 (Visit 1)—infusion of drugs to volunteers according to group allocation; Day 2 (Visit 2)—hospital discharge; Days 3 through 14 (Visits 3–5)—outpatient observation of the volunteer; 4. Period II (Day 15–Day 28) including: Day 15 (Visit 6)—cross-over infusion of the drug to volunteers according to group allocation; Day 16 (Visit 7)—hospital discharge; Days 17 through 28 (Visits 8–10)—outpatient observation of the volunteer; 5. Study Completion—Day 29 (Visit 11). The duration of one volunteer’s participation in the clinical study was up to 43 days (see Figure 4).

#### 3.4.2. Pharmacokinetics

The time course of agalsidase β in the GNR-062 and Fabrazyme^®^ groups is shown in Figure 5. Table 3, Table 4 and Table 5 summarize the background characteristics of the participants in the study and the pharmacokinetic parameters, respectively.

One volunteer out of 36 (2.8%) withdrew informed consent on Day 7 and terminated the study early (Table 4). The remaining 35 volunteers completed the study according to the protocol.

The arithmetic mean value of the area under the concentration-time curve in the time interval from 0 to infinity (AUCinf) of the drug GNR-062 was 510,301.0 ± 134148.3 ng· min/mL while for Fabrazyme^®^ it was 520,146.6 ± 105,503.3 ng·min/mL, *p* = 0.635. The geometric mean AUCinf values of GNR-062 and Fabrazyme^®^ were 493,859.8 ng·min/mL and 510,381.6 ng·min/mL, respectively. The arithmetic mean value of the area under the concentration-time curve in the interval from 0 to the time of the last measurable concentration (AUC0-t) of the drug GNR-062 was 508,532.9 ± 133,507.1 ng·min/mL; the drug Fabrazyme^®^—518,037.2 ± 105,218.8 ng·min/mL, *p* = 0.670. The geometric mean AUC0-t values of the drugs GNR-062 and Fabrazyme^®^ were 492,193.4 ng·min/mL and 508,289.5 ng·min/mL, respectively. The arithmetic mean maximum drug concentration (Cmax) of GNR-062 was 1748.71 ± 456.68 ng/mL; the drug Fabrazyme^®^—1763.28 ± 375.86 ng/mL, *p* = 0.793. The geometric mean Cmax values of the drugs GNR-062 and Fabrazyme^®^ were 1692.61 ng/mL and 1726.26 ng/mL, respectively. The analysis confirmed the bioequivalence of the two drugs for all main PK parameters, since the 90% CI for the ratio of PK parameters was completely within the range of 0.80–1.25, which indicated the equivalence of the test and reference drugs for the main PK parameters (Table 5 and Table 6).

The infusion was administered over 300 min. The median Tmax of 180 min for both treatments was observed during the infusion, which was consistent with the sampling schedule and the pharmacokinetic profile of intravenously administered agalsidase β. The maximum concentration was reached before the end of the infusion, and concentrations remained elevated until the end of infusion, after which they declined monophasically.

#### 3.4.3. Safety

The main safety parameters were the number of volunteers experiencing adverse events (during the entire study period from the start of the infusion to the end of the study (Day 29); the frequency, severity, and causality of AEs, and the cause-and-effect relationship of the occurrence of AEs with the use of study drugs during the study period based on subjective complaints, physical examination, assessment of vital signs, and laboratory and instrumental studies in volunteers in the comparison groups. The study recorded 46 adverse events (AEs) in 17 volunteers: 10 after GNR-062 administration and 7 after Fabrazyme^®^ administration. All AEs were mild and unrelated to the study drugs. AEs were summarized below by system organ class (SOC) and preferred term (PT) according to MedDRA (Table 7).

Serious adverse events (AEs) during the study were not reported. Of the 46 AEs reported, 40 had not resolved at the end of the formal study period (Day 29). All participants with unresolved abnormalities were followed until resolution or stabilization. At the final follow-up, they remained stable and clinically insignificant.

#### 3.4.4. Immunogenicity

A bioanalytical study was conducted to determine the content and characterization of anti-drug antibodies (ADA) to the drug GNR-062 in 108 blood serum samples taken from 36 volunteers of a clinical trial. All tested samples were free of ADA: according to the results of the screening test for the presence of ADA, 16 conditionally positive samples were detected, of which 16 samples were not confirmed by the results of the confirmatory test. Due to the lack of confirmed positive samples ADA, analysis for the presence of neutralizing ADA and IgE to agalsidase β was not performed.

## 4. Discussion

Fabry disease is caused by a deficiency in the lysosomal enzyme α-galactosidase A; therefore, effective treatment requires the delivery of a functional recombinant analogue into the cells of patients. This intracellular targeting is mediated by the mannose-6-phosphate (M6P) receptor, which is responsible for the sorting and transport of lysosomal enzymes. We have developed a new recombinant agalsidase β (GNR-062) expressed in a Chinese hamster ovary (CHO) cell line using established genetic engineering methods, analogous to the process for Fabrazyme^®^. It is a glycosylated homodimer with a molecular mass of approximately 51 kDa per subunit and exhibits significant microheterogeneity in its glycan modifications. Its overall chemical composition and structure are similar to those of the reference product. Direct HPLC analysis revealed differences primarily in the distribution profiles of complex sialylated N-glycans, although the overall degree of sialylation was comparable between the two products; this finding was corroborated by the sialic acid content analysis. Furthermore, despite a minor difference (a few percentage points) in the level of bisphosphorylated glycoforms, the total measured M6P content was considered comparable. Evaluation of the in vitro pharmacological properties showed no notable differences: the products demonstrated comparable efficiency in penetrating fibroblasts derived from a Fabry disease patient, as well as equivalent levels of enzymatic activity. These in vitro data provided the rationale for proceeding to a first-in-human clinical study. This first-in-human study was designed as a single-blind, randomized, crossover comparative study in healthy male volunteers to evaluate the pharmacokinetic (PK) parameters and safety of GNR-062. Participants were blinded to treatment assignment, whereas investigators were not; laboratory personnel performing PK and immunogenicity assessments were also blinded to sample identity. In this study, the geometric mean ratio of the Cmax value for α-galactosidase A between GNR-062 and Fabrazyme^®^ at 90% CI was 0.9867 (0.9546, 1.0199). AUCinf and AUC in the GNR-062 group were slightly lower than in the Fabrazyme^®^ group but remained within the bioequivalence range (80–125%), while the T1/2 of α-galactosidase A was similar for GNR-062 and Fabrazyme^®^, consistent with the in vitro biological properties (enzyme activity and cellular uptake) studies. It is known that M6P residues on recombinant agalsidase β or α bind to the plasma membrane via the M6P receptor and are internalized to the site of action in cellular lysosomes. Once transported into lysosomes, tissue uptake is effectively irreversible, with no release back into the bloodstream, owing to the efficiency of the M6P receptor in sequestering this enzyme within the lysosome [28]. Since most mammalian somatic cells express the M6P receptor and GNR-062 contains M6P residues, GNR-062 would be expected to be widely distributed in tissues after intravenous administration [29]. Furthermore, the major mechanism of agalsidase clearance from serum is M6P-mediated intracellular uptake. In our study using fibroblasts from patients with Fabry disease, we did not observe differences in cellular uptake or enzymatic activity between the two products, possibly owing to the variability inherent in cell-based assays. In contrast, the literature data indicate a more pronounced difference in M6P content, receptor binding, and cellular internalization (more than 1.5-fold); however, this difference did not affect the pharmacokinetic results [19,21,30]. In clinical studies of agalsidase β, adverse reactions such as chills and fever were frequently reported. Moreover, when recombinant α-Gal A was administered to patients with Fabry disease, one of the major concerns was the formation of antibodies to recombinant α-Gal A [7]. However, none of the healthy subjects in this single-dose study experienced any reactions during the entire infusion period, and no infusion interruption or change in infusion rate was required. Recombinant agalsidase β (GNR-062) was well tolerated by all study participants, and none experienced an immunogenic response to the drug. The absence of an immune response may be explained by two factors: first, only a single dose was administered to healthy subjects; second, the administered protein, agalsidase β, might not be regarded as foreign in healthy subjects because it is constitutively produced in the body. The absence of infusion reactions, in turn, is consistent with the overall lack of immunogenicity observed in this single-dose setting. Throughout the study, no AEs related to the study drug were observed. Furthermore, the crossover design used in our study allowed us to evaluate the effect of switching from one drug to another. This first-in-human study was designed to evaluate the PK parameters and safety of the new recombinant agalsidase β (GNR-062), providing the basis for clinical trials in Fabry patients to further evaluate the safety and efficacy of this new recombinant drug. This study has several limitations. First, only healthy male volunteers aged 18–41 years with a body weight of 50–70 kg were enrolled. This population does not represent women, older individuals, participants with broader body weights, or patients with Fabry disease, in whom disease-related organ dysfunction and pre-existing immune status may affect pharmacokinetics, safety, and immunogenicity. Second, the single-dose design does not reflect repeated long-term administration and therefore precludes assessment of immunogenicity upon repeated dosing. Third, the study was single-blind for participants but open-label for investigators, which represents a potential source of bias. To mitigate this risk, laboratory personnel were blinded to the samples: all samples for immunogenicity and pharmacokinetics assessment were processed and analyzed by laboratory personnel unaware of the treatment group assignments. Nonetheless, the absence of investigator blinding is a limitation that should be taken into account when interpreting safety and tolerability data.

## 5. Conclusions

The present study demonstrated structural and functional similarity between GNR-062 and Fabrazyme^®^, as well as pharmacokinetic bioequivalence. GNR-062 was not associated with any new adverse events compared with the existing α-galactosidase A formulation and was generally well tolerated after a single dose in healthy male volunteers. However, given the small sample size, single-dose exposure, and the absence of patients with Fabry disease, these findings are limited to short-term tolerability. GNR-062 may warrant further investigation as a potential therapeutic option for patients with Fabry disease.

## Figures and Tables

**Figure 1:**
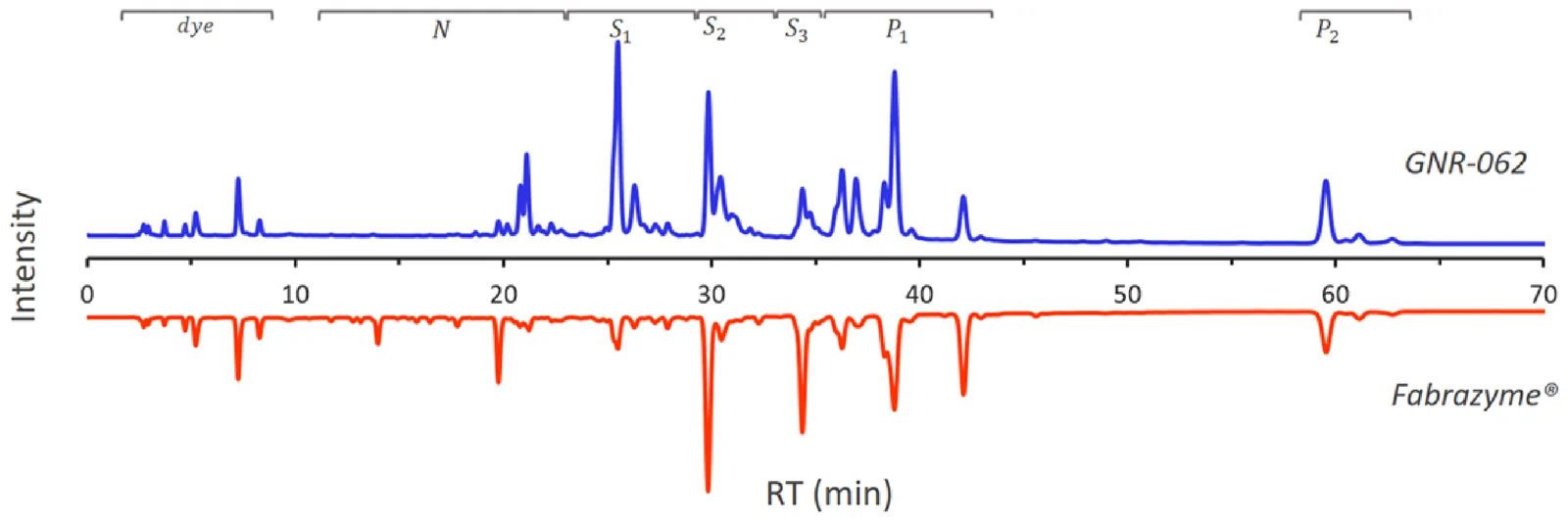
A mirror plot n example glycosylation profiles of GNR-062 and Fabrazyme^®^ glycosylation profiles.^®^.

**Figure 2:**
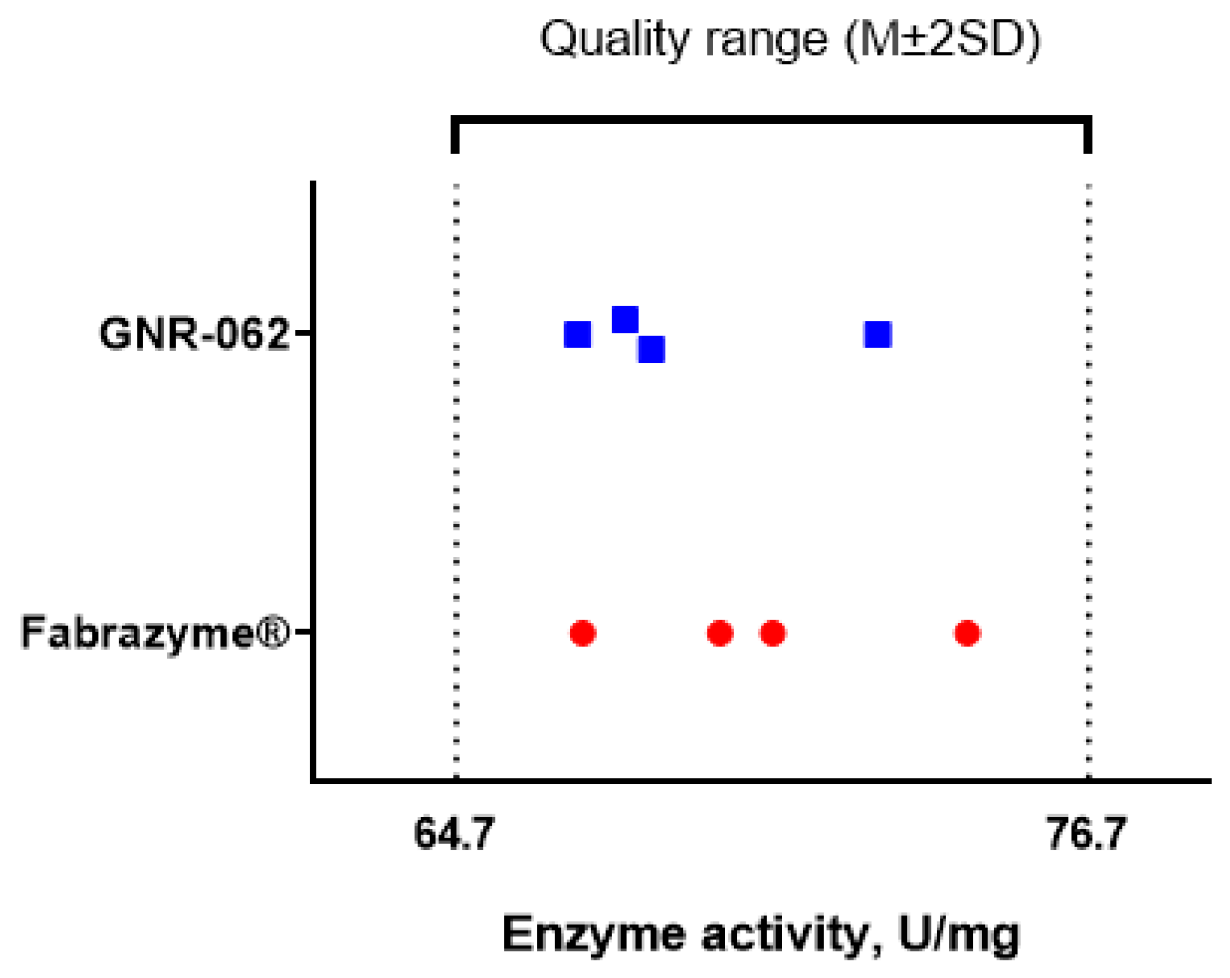
Enzyme activity of GNR-062 and Fabrazyme^®^.pNP-α-D-Gal was used as a substrate. Results from representative lots of GNR-062 and Fabrazyme^®^ are shown.

**Figure 3:**
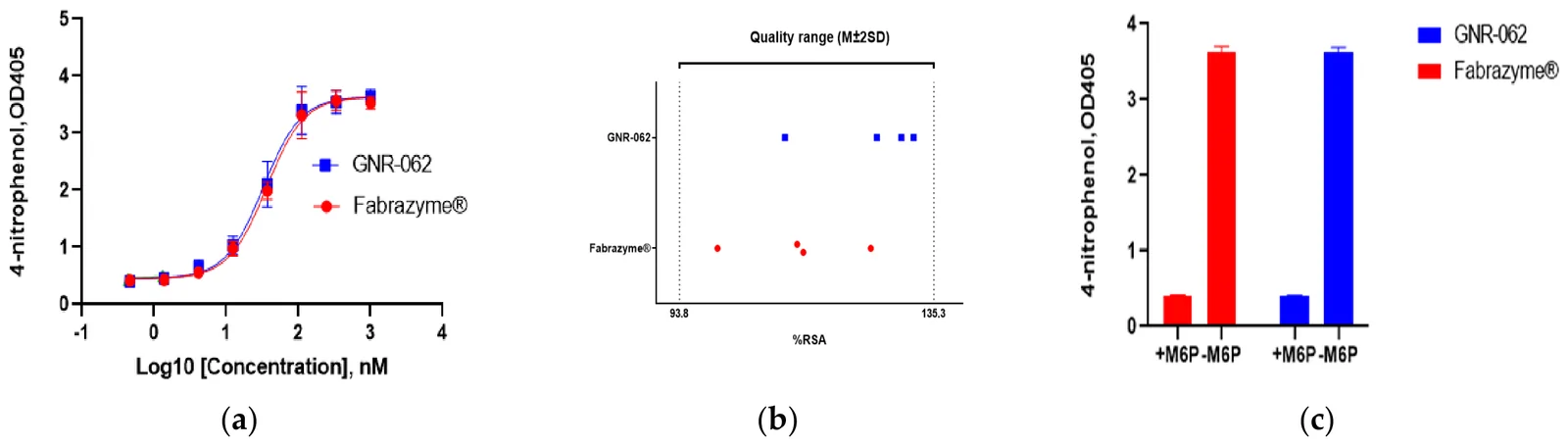
Cellular uptake of agalsidase β. (**a**) Curve uptake of GNR-062 and Fabrazyme^®^ into cells. Results from batch-level data. (**b**) Results of the relative specific activity of the products following cellular uptake. (**c**) An example M6PR-dependent uptake of GNR-062 and Fabrazyme^®^ into cells.

**Figure 4:**
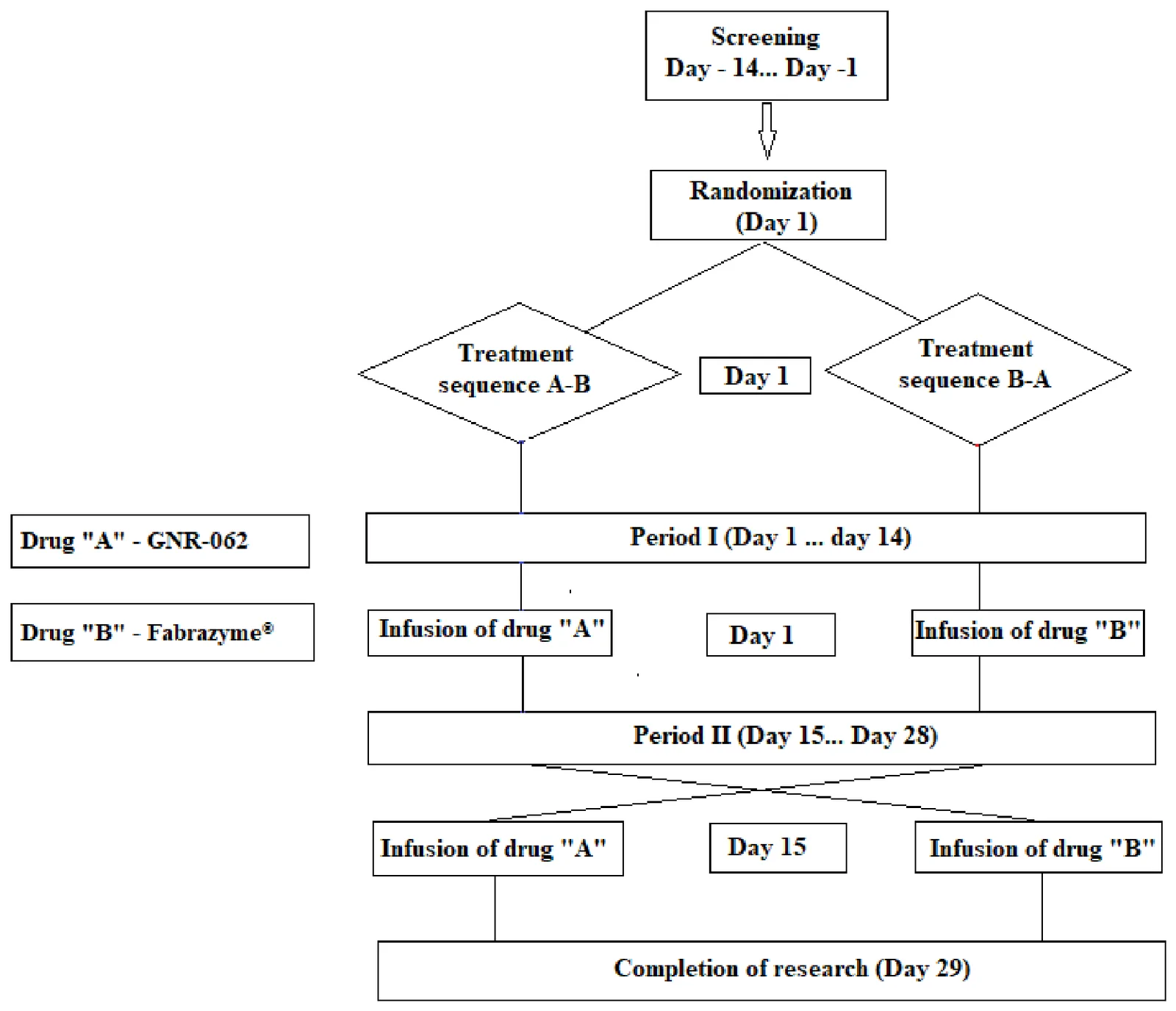
Distribution of volunteers.

**Figure 5:**
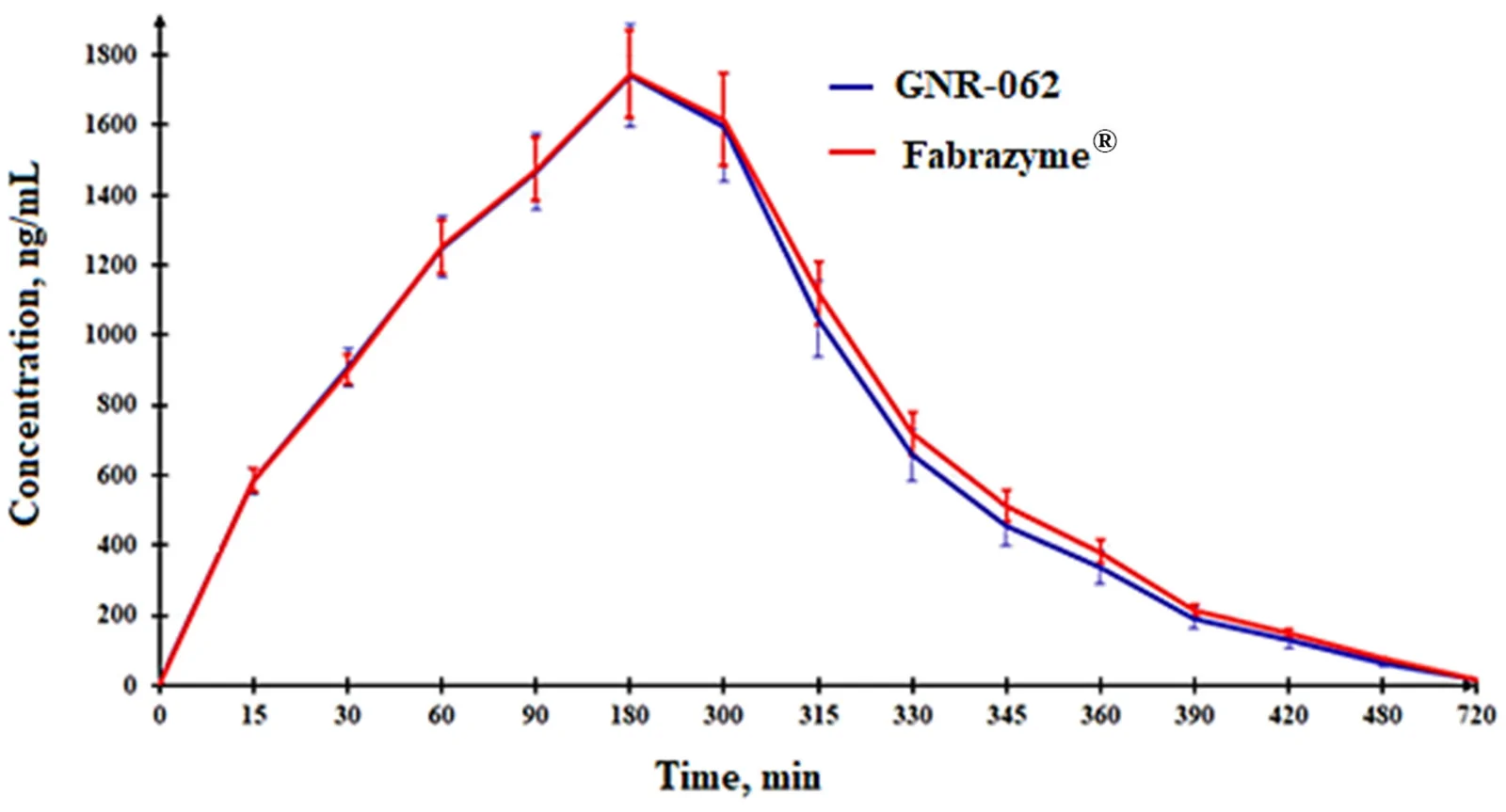
Time course changes in sera α-galactosidase A concentration. Red line, Fabrazyme^®^ group, GNR-062 blue line; error bars show the 95% confidence interval.

**Table 1:** N-glycan distribution (%).

DP	Fabrazyme^®^	GNR-062
N	11.7 ± 0.5	10.6 ± 0.3
S1	7.1 ± 0.3	20.3 ± 2.1
S2	19.3 ± 0.4	18.3 ± 1.4
S3	12.6 ± 0.1	6.2 ± 1.2
P1	37.0 ± 0.4	35.6 ± 1.7
P2	12.3 ± 0.5	9.0 ± 0.7

**Table 3:** Background characteristics of the participants in the Phase I study (FAS population).

Index	Parameter	Group I (GNR-062/Fabrazyme^®^)	Group II (Fabrazyme^®^/GNR-062)	Total	*p*
Age, years	*N*	18	18	36	0.770
	M	28.2	27.6	27.9	
	SD	7.9	5.4	6.7	
	95% CI	(24.3; 32.2)	(24.9; 30.3)	(25.6; 30.2)	
	Min	18	21	18	
	Max	41	41	41	
	Me	25	26.5	25.5	
	Q1	23	24	23.5	
	Q3	37	30	33	
Height, cm	*N*	18	18	36	0.054
	M	176.0	179.6	177.8	
	SD	5.8	5.1	5.7	
	95% CI	(173.1; 178,9)	(177.1; 182.1)	(175.9; 179.7)	
	Min	165	169	165	
	Max	185	188	188	
	Me	175.5	180	178.5	
	Q1	172	176	173	
	Q3	180	183	182.5	
Weight, kg	*N*	18	18	36	0.620
	M	65.45	66.15	65.80	
	SD	4.81	3.47	4.15	
	95% CI	(63.06; 67.84)	(64.43; 67.87)	(64.4; 67.20)	
	Min	55.6	57.4	55.6	
	Max	69.5	69.6	69.6	
	Me	68.2	66.5	67.7	
	Q1	61.9	64.0	63.5	
	Q3	69.4	69.3	69.4	
Body mass index, kg/m^2^	*N*	18	18	36	0.255
	M	21.14	20.54	20.84	
	SD	1.63	1.51	1.58	
	95% CI	(20.34; 21.95)	(19.79; 21.29)	(20.31; 21.38)	
	Min	18.6	18.5	18.5	
	Max	23.8	22.8	23.8	
	Me	20.9	20.6	20.8	
	Q1	20.2	19.1	19.6	
	Q3	22.3	22.0	22.0	

**Table 4:** Distribution of study subjects.

Volunteer Distribution	Number of Volunteers Share (%)
Screened	49/49 (100.0)
Volunteers who do not meet inclusion/exclusion criteria Included in the study (FAS):	13/49 (26.5)
Included in the study (FAS): • Group I (GNR-062/Fabrazyme^®^) • Group II (Fabrazyme^®^/GNR-062)	36/49 (73.5): • 18/36 (50.0) • 18/36 (50.0)
Population for safety analysis	36/36 (100.0)
Dropped out prematurely	1/36 (2.8)
Completed the study according to the protocol	35/36 (97.2)
Volunteers for evaluation of PK parameters:	36/36 (100.0)

**Table 5:** Pharmacokinetic parameters in the Phase I study.

**Parameter**	**AUC, ng·min/mL**		**AUCinf, ng·min/mL**		**Cmax, ng/mL**		**Tmax, min**	
	**GNR-062**	**Fabrazyme^®^**	**GNR-062**	**Fabrazyme^®^**	**GNR-062**	**Fabrazyme^®^**	**GNR-062**	**Fabrazyme^®^**
*N*	36	35	36	35	36	35	36	35
M	508,532.9	518,037.2	510,301.0	520,146.6	1748.71	1763.28	196.7	210.9
SD	133,507.1	105,218.8	134,148.3	105,503.3	456.68	375.86	42.1	53.2
gMean	492,193.4	508,289.5	493,859.8	510,381.6	1692.613	1726.26	193.2	205.3
95% CI for gMean	(451,102.0; 537,027.8)	(475,157.3; 543,732.0)	(452,573.4; 538,912.6)	(477,147.9; 545,930.0)	(1551.03; 1847.12)	(1607.06; 1854.29)	(181.9; 205.3)	(189.9; 221.9)
Min	336,807.8	347,460.0	337,619.9	349,309.7	1097.5	1201.2	180	180
Max	782,891.1	868,091.4	786,152.8	870,592.7	2672.5	2997.1	300	300
Me	451,217.8	512,917.7	452,368.7	515,506.6	1616.4	1752.2	180	180
Q1	398,917.7	453,848.3	399,970.7	455,523.4	1329.3	1528.6	180	180
Q3	628,900.1	592,885.3	631,372.8	595,323.3	2131.6	2012.5	180	300
CV, %	26.3%	20.3%	26.3%	20.3%	26.1%	21.3%	21.4%	25.2%
p (Wilcoxon)	0.670		0.635		0.793		0.0455	
**Parameter**	**T1/2, min**		**Kel, min^−1^**		**CL, mL/min**		**Vd, L**	
	**GNR-062**	**Fabrazyme^®^**	**GNR-062**	**Fabrazyme^®^**	**GNR-062**	**Fabrazyme^®^**	**GNR-062**	**Fabrazyme^®^**
*N*	36	35	36	35	36	35	36	35
M	88.04	88.46	0.0079	0.0079	136.92	131.52	17.46	16.81
SD	4.45	8.27	0.0004	0.0011	32.81	25.70	4.56	3.84
gMean	87.93	87.99	0.0079	0.0079	133.14	129.15	16.89	16.39
95% CI for gMean	(86.46; 89.42)	(84.71; 91.40)	(0.0078; 0.0080)	(0.0076; 0.0082)	(122.72; 144.44)	(120.85; 138.02)	(15.46; 18.46)	(15.16; 17.73)
Min	81.1	50.8	0.0070	0.0069	87.6	77.0	10.4	8.8
Max	99.5	100.1	0.0085	0.0136	207.3	200.4	29.1	27.3
Me	87.7	89.0	0.0079	0.0078	134.0	127.1	16.8	16.1
Q1	85.5	86.0	0.0077	0.0076	107.6	113.6	13.5	13.9
Q3	89.9	91.1	0.0081	0.0081	160.0	142.7	20.4	19.1
CV, %	5.1%	9.3%	4.9%	13.6%	24.0%	19.5%	26.1%	22.8%
p (Wilcoxon)	0.0011		0.0008		0.067		0.658	

**Table 6:** Comparison of Pharmacokinetic Parameters Between GNR-062 and Fabrazyme^®.^

Parameter	Comparison of Parameters Between GNR-062 and Fabrazyme^®^			*p*
	Point Estimate	90% CI		
AUC	0.9743	0.9421	1.0076	0.203
AUCinf	0.9736	0.9413	1.0070	0.191
Cmax	0.9867	0.9546	1.0199	0.507

**Table 7:** Adverse events reported during the analysis period, by organ system class, preferred term, and treatment group (Safety Population).

Description	GNR-062 *N* = 36		Fabrazyme^®^ *N* = 35 *		Total *N* = 36	
	*N*	%	*N*	%	*N*	%
Preferred term	10	27.8	7	20.0	17	47.2
Laboratory and instrumental data	10	27.8	7	20.0	17	47.2
Increased the number of basophils	7	19.4	2	5.7	9	25.0
Decreased hemoglobin levels	4	11.1	3	8.6	7	19.4
Decreased hematocrit indicator	4	11.1	1	2.9	5	13.9
Increasing phosphorus levels in the blood	2	5.6	1	2.9	3	8.3
Increased number of eosinophils	1	2.8	2	5.7	3	8.3
Decreased mean corpuscular hemoglobin	2	5.6	1	2.9	3	8.3
Increased lymphocyte count	2	5.6	1	2.9	3	8.3
Decreased mean corpuscular volume	2	5.6	1	2.9	3	8.3
Increased uric acid levels in the blood	1	2.8	1	2.9	2	5.6
Decreased red blood cell count	2	5.6	0	0.0	2	5.6
Increased leukocyte count	1	2.8	0	0.0	1	2.8
Increased monocyte count	1	2.8	0	0.0	1	2.8
Increased neutrophil count	1	2.8	0	0.0	1	2.8
Increased platelet count	0	0.0	1	2.9	1	2.8
Decreased leukocyte count	0	0.0	1	2.9	1	2.8
Shortening of the QT interval on the electrocardiogram	0	0.0	1	2.9	1	2.8

Notes: Data are presented as the number and percentage of subjects in the safety population who experienced AEs after receiving the drug. *—Informed consent withdrawn by the volunteer on Day 7 of the study.

## Data Availability

The original contributions presented in this study are included in the article. Further inquiries can be directed to the corresponding author.

## Abbreviations

Abbreviations used in this manuscript:

ADA	anti-drug antibodies
AUC0-∞ (AUCinf)	area under the curve extrapolated to infinity
AUC0-t	area under the curve from time 0 to last measurable time point
CHO	Chinese hamster ovary
95% CI	95% confidence interval
CL	total body clearance
Cmax	maximum serum concentration
EC_50_	half-maximal effective concentration
FAS	full analysis set
h	hour
kDa	kilodalton
Kel	elimination rate constant
L	liter
L/h	liter per hour
LLOQ	lower limit of quantification
M	mean
M6P	mannose-6-phosphate
M6PR	mannose-6-phosphate receptor
Max	maximum
Me	median
MedDRA	Medical Dictionary for Regulatory Activities
Min	minimum
µL	microliter
*N*	number of subjects
NAb	neutralizing antibodies
Neu5Ac	N-acetylneuraminic acid
Neu5Gc	N-glycolylneuraminic acid
ng/mL	nanograms per milliliter
OD	optical density unit
*p*	*p*-value
PK	pharmacokinetics
Q1	quartile 1
Q3	quartile 3
QC	quality control
QR	quality range
RLD	reference listed drug
RP	reference product
RSA	relative specific activity
SD	standard deviation
T1/2	elimination half-life
Tmax	time to reach maximum serum concentration
TMB	3,3′,5,5′ tetramethylbenzidine
Vd	volume of distribution
